# Impact of diabetes mellitus and body mass index on long-term survival in chronic total occlusion patients: a nationwide cohort study from the SCAAR registry

**DOI:** 10.1136/bmjopen-2025-100074

**Published:** 2025-09-17

**Authors:** Mohammed Mohammed, Joakim Sundström, Antros Louca, Gustaf Hellsen, Aidin Rawshani, Göran K Olivecrona, Moman Aladdin Mohammad, Dan Ioanes, Ulf Jensen, David Erlinge, Oskar Angerås, Petur Petursson, Anna Myredal, Sebastian Völz, Christian Dworeck, Jacob Odenstedt, Araz Rawshani, Truls Råmunddal

**Affiliations:** 1 Department of Molecular and Clinical Medicine, University of Gothenburg Sahlgrenska Academy, Gothenburg, Sweden; 2 Department of Cardiology, Sahlgrenska University Hospital, Gothenburg, Sweden; 3 Department of Internal Medicine, Sahlgrenska Universitetssjukhuset Östra sjukhuset, Gothenburg, Sweden; 4 Department of Cardiology, Department of Clinical Sciences, Lund University, Lund, Sweden; 5 Department of Clinical Science and Education, Karolinska Institute, Stockholm, Sweden

**Keywords:** Body Mass Index, Cardiovascular Disease, DIABETES & ENDOCRINOLOGY, Coronary heart disease, Ischaemic heart disease, General diabetes

## Abstract

**Abstract:**

**Objectives:**

To evaluate the effects of diabetes mellitus (DM) and body mass index (BMI) on long-term all-cause mortality in chronic total occlusion (CTO) patients.

**Design:**

Retrospective, nationwide cohort study.

**Setting:**

Swedish Coronary Angiography and Angioplasty Registry, between June 2015 and December 2021.

**Participants:**

24 284 patients with angiographically confirmed CTO. Prior coronary artery bypass graft surgery excluded. Subgroups were defined by DM status and BMI categories (underweight, healthy weight, overweight, obesity).

**Primary outcome measures:**

Long-term all-cause mortality, assessed by Kaplan-Meier analysis and multivariable Cox proportional hazards regression.

**Results:**

DM was present in 30.3% of patients and conferred a 31% higher risk of mortality (HR: 1.31, 95% CI: 1.20 to 1.42; p<0.001). Insulin use among patients with diabetes added a 52% increase in hazard (HR: 1.52; 95% CI: 1.38 to 1.67; p<0.001). BMI demonstrated a non-linear association with mortality: overweight (HR: 0.70, 95% CI: 0.64 to 0.77; p<0.001) and obese (HR: 0.74, 95% CI: 0.68 to 0.81; p<0.001) groups had lower risk compared with the healthy-weight group, whereas underweight individuals faced the highest risk (HR: 1.61, 95% CI: 1.25 to 2.08; p<0.001). A continuous BMI spline revealed an asymmetric U-shaped association: a steep increase in mortality below 23 kg/m^2^, lowest risk (nadir) at 32 kg/m^2^ and modest rise above 35 kg/m^2^.

**Conclusions:**

In this nationwide CTO cohort, DM independently predicted higher long-term mortality, accompanied by more severe comorbidities and greater CTO complexity, and insulin therapy further elevated hazard. Overweight and obese patients had better survival, while underweight individuals had the poorest prognosis. These findings underscore the importance of individualised risk assessment and management strategies in CTO patients, particularly those with DM or low BMI.

**Strengths and limitations of this study**

- Nationwide, large Swedish Coronary Angiography and Angioplasty Registry cohort with consecutive enrolment and >99% mortality follow-up minimises regional selection and attrition bias, boosting generalisability.
- Rich clinical detail (200 baseline and procedural variables, including insulin-use status) permits extensive confounder adjustment and nuanced analyses of diabetes mellitus and body mass index.
- Transparent missing-data strategy: excluding variables with >30% missingness and applying fully documented multiple imputation—reduces bias and supports reproducibility.
- Observational design caveat: residual confounding cannot be ruled out, and non-adjudicated deaths prevent clear separation of cardiac versus non-cardiac mortality.
- Key data gaps: no diabetes duration, haemoglobin A1c (HbA1c), insulin duration/type, body composition or cardiorespiratory-fitness measures, potentially misclassifying metabolic status and obesity phenotypes.

## Introduction

 Chronic total occlusion (CTO) represents a particularly challenging subset of coronary artery disease (CAD). It is defined as a complete obstruction of an epicardial coronary artery with the absence of anterograde flow persisting for at least 3 months.^1 2^ CTO is observed in 16%–30% of patients undergoing coronary angiography and nearly one-fourth of those with chronic coronary syndrome.^1 2^

The presence of CTO is associated with increased mortality risk, especially in younger patients and those with acute coronary syndrome.^2^

A major contributor to vascular complications is diabetes mellitus (DM), which is characterised by persistent hyperglycaemia, β-cell dysfunction and insulin resistance. DM accelerates the progression of atherosclerosis, resulting in more extensive and severe coronary artery lesions. Cardiovascular disease accounts for 65%–75% of deaths among individuals with diabetes.^3^,^5^

Body mass index (BMI) is another critical determinant of cardiovascular outcomes in CAD patients, with the prevalence of obesity increasing significantly in the population. Although obesity is typically associated with an increased prevalence of cardiovascular risk factors such as hypertension and DM, several studies suggest that moderate overweight or even obesity may have protective effects in some CAD cases, a phenomenon known as the ‘obesity paradox’.^6^,^8^

To further elucidate the relationship between DM, BMI and CAD, this study investigates the prognostic impact of DM and BMI on long-term survival in CTO patients, using data from Swedish Coronary Angiography and Angioplasty Registry (SCAAR).

## Methods

This study is reported according to the Strengthening the Reporting of Observational Studies in Epidemiology guidelines.

### Study design and setting

A retrospective, nationwide cohort study was conducted using data obtained from the SCAAR, which is part of the national Swedish Web-System for Enhancement and Development of Evidence-Based Care in Heart Disease Evaluated According to Recommended Therapies (SWEDEHEART) registry. SCAAR contains detailed records of all coronary angiography and percutaneous coronary intervention (PCI) procedures in Sweden. It is fully funded by the Swedish Health Authorities and managed by the Uppsala Clinical Research Center. Since 2001, SCAAR has used a web-based reporting platform with automatic data monitoring, involving 30 hospitals, including 9 university hospitals. Procedures are detailed with around 50 variables for coronary angiography and 200 for PCI, entered by PCI physicians after review. All entries undergo automated range and consistency checks at the time of reporting. Survival data are continuously updated through the national death registry, though specific causes of death are not verified.^2 8 9^ The study period was 2 June 2015 to 31 December 2021.

### Participants

**Figure 1:**
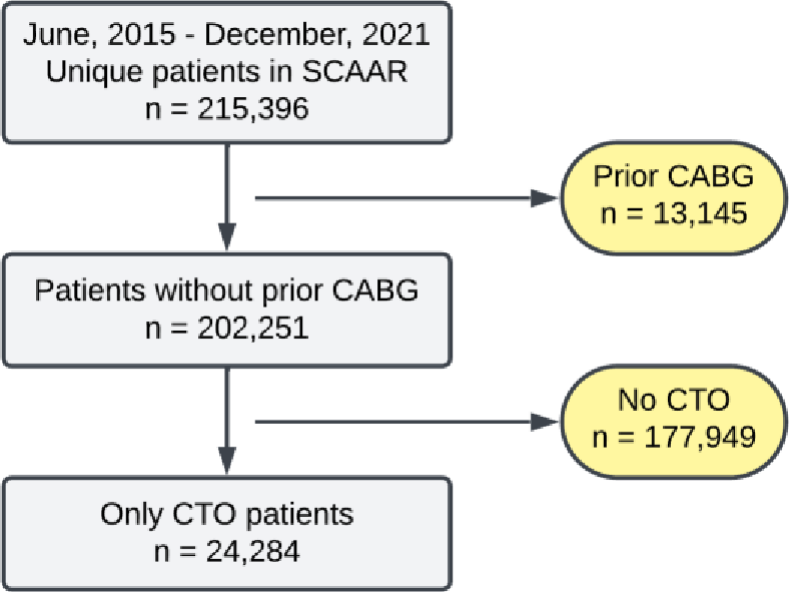
Flow chart for patient inclusion. CABG, coronary artery bypass grafting; CTO, chronic total occlusion; SCAAR, Swedish Coronary Angiography and Angioplasty Registry.

Eligible participants were all patients with angiographically confirmed CTO who underwent coronary angiography during the study period (figure 1). CTO was defined as a complete coronary artery obstruction persisting for at least 3 months. Exclusion criteria comprised prior coronary artery bypass graft surgery (CABG) due to inability to evaluate graft patency, absence of documented CTO lesions, negative survival times and implausible ages (< 0 or > 120 years). The final analytical cohort consisted of 24 284 unique CTO patients. Because this analysis captured all eligible CTO patients in Sweden during the study period, no formal sample size calculation was performed.

### Definitions and variables

#### Outcome

The primary outcome was all-cause mortality, determined via the national death registry. Time to event was calculated from the date of the index angiography to the date of death or administrative censoring at 31 December 2021.

#### Exposures

DM was coded as yes/no in the registry; ‘unknown’ entries were treated as missing (NA). BMI was calculated as weight (kg) divided by height (m)^2^ and categorised according to WHO criteria: underweight <18.5 kg/m², normal weight 18.5–24.9 kg/m², overweight 25.0–29.9 kg/m² and obesity ≥30 kg/m².^10^ Insulin use among patients with DM was recorded and used in stratified analyses.

#### Covariates

Prespecified covariates included age (≤59, 60–79, ≥80 years), sex, smoking status (never, former, current), hypertension, hyperlipidaemia, previous myocardial infarction (MI), previous PCI, clinical indication for angiography (stable CAD, unstable CAD/non-ST-elevation MI (NSTEMI), STEMI, cardiac arrest, other) and CTO burden (1, 2, ≥3 CTO vessels).

Other baseline variables included estimated glomerular filtration rate (eGFR), calculated from serum creatinine using the Chronic Kidney Disease Epidemiology Collaboration equation without race adjustment; eGFR was reported descriptively.

### Data sources and measurement

All variables were obtained from SCAAR. Variable coding followed registry definitions. Where present, factors labelled ‘unknown’ (code 9) were recoded to missing, ensuring that missing entries were not misclassified as valid categories. CTO definitions followed registry standards and prior SCAAR publications.

### Bias and study size

National coverage and linkage to the death registry minimised selection and attrition bias. To limit information bias, variables with >30% missingness were excluded from multivariable Cox regression to preserve statistical validity and avoid unreliable imputations. No formal sample size calculation was performed because all eligible CTO patients in Sweden during the study window were included.

### Handling of quantitative variables

Age was analysed in prespecified categories; eGFR was reported descriptively only. BMI was analysed both as categories and as a continuous exposure using restricted cubic splines with three knots; curves were centred at BMI 25 kg/m^2^.

### Missing data

Patterns of missingness were systematically evaluated using the naniar package, and proportions of missing values were included in the baseline tables (tables1^2^). Remaining missing values for covariates and exposures used in modelling were addressed using multiple imputation assuming data were Missing at Random, by chained equations with random-forest imputation (mice package, seed 2023, m=5, maxit=10, method=‘rf’). Categorical predictors were imputed as factors after setting clinically meaningful reference levels. Descriptive summaries are based on observed data. Cox regression estimates were obtained from the imputed datasets and combined using Rubin’s rules; complete-case analyses served as sensitivity checks.

**Table 1:** Baseline characteristics of patients stratified by diabetes mellitus (DM)

	No DM	DM†	P value	Missing	SMD
	(n=16 800)	(n=7352)			
Gender			0.002	0	0.04
Female; n (%)	3484 (20.7)	1659 (22.6)			
Male; n (%)	13 316 (79.3)	5693 (77.4)			
Age median; IQR (years)	71.00 (63.00–77.00)	71.00 (64.00–76.00)	0.235	0	0.01
Age			<0.001	0	0.14
<59; n (%)	2957 (17.6)	1109 (15.1)			
60–79; n (%)	10 736 (63.9)	5167 (70.3)			
>80; n (%)	3107 (18.5)	1076 (14.6)			
Height median; IQR (cm)	175.00 (168.00–180.00)	174.00 (168.00–180.00)	<0.001	4111 (16.9%)	0.07
Weight median; IQR (kg)	81.00 (72.00–92.00)	86.00 (76.00–98.00)	<0.001	3414 (14.1%)	0.30
BMI median; IQR (kg/m²)	26.78 (24.38–29.74)	28.68 (25.76–32.00)	<0.001	4175 (17.2%)	0.29
Diabetes insulin usage; n (%)	0	3331 (45.7)	<0.001	71 (0.30%)	1.30
Smoking status			<0.001	1443 (5.9%)	0.12
Current smoker; n (%)	2991 (18.8)	1167 (17.0)			
Non-smoker; n (%)	6358 (39.9)	2479 (36.1)			
Previous smoker; n (%)	6569 (41.3)	3229 (47.0)			
Extent of CTO			<0.001	0	0.11
1 CTO; n (%)	14 017 (83.4)	5831 (79.3)			
2 CTO; n (%)	2285 (13.6)	1239 (16.9)			
three or more CTOs; n (%)	498 (3.0)	282 (3.8)			
Hypertension; n (%)	11 292 (67.7)	6457 (88.5)	<0.001	255 (1.1%)	0.52
Hyperlipidaemia; n (%)	9125 (54.8)	5588 (77.0)	<0.001	326 (1.3%)	0.48
Previous MI; n (%)	4725 (28.8)	2850 (39.9)	<0.001	669 (2.8%)	0.24
Indication for angiography			<0.001	111 (0.5%)	0.12
Stable CAD; n (%)	4748 (28.4)	1986 (27.1)			
Unstable CAD/NSTEMI; n (%)	6581 (39.4)	3193 (43.6)			
STEMI; n (%)	2470 (14.8)	841 (11.5)			
Cardiac arrest without STEMI; n (%)	228 (1.4)	120 (1.6)			
Cardiac arrest with STEMI; n (%)	158 (0.9)	66 (0.9)			
Other; n (%)	2539 (15.2)	1111 (15.2)			
CCS class			0.038	18 (0.07%)*	0.08
I; n (%)	619 (13.1)	236 (11.9)			
II; n (%)	2667 (56.3)	1069 (54.0)			
III; n (%)	1401 (29.6)	655 (33.1)			
IV; n (%)	49 (1.0)	20 (1.0)			
eGFR median; IQR (mL/min/1.73 m²)	77.90 (62.30–89.20)	73.60 (54.02–89.20)	<0.001	5170 (21.3%)	0.20

* Calculated within the ‘stable CAD’ group only.

† DM status was missing for 132 observations.

BMI, body mass index; CAD, coronary artery disease; CCS, Canadian Cardiovascular Society; CTO, chronic total occlusion; eGFR, estimated glomerular filtration rate; MI, myocardial infarction; n, sample size; NSTEMI, non-STEMI; SMD, standardised mean difference; STEMI, ST-elevation myocardial infarction.

**Table 2:** Baseline characteristics of patients stratified by body mass index (BMI)

	Underweight	Healthy weight	Overweight	Obesity	P value	Missing	SMD
	(n=146)	(n=5564)	(n=8736)	(n=5663)			
Gender					<0.001	0	0.47
Female; n (%)	85 (58.2)	1377 (24.7)	1497 (17.1)	1257 (22.2)			
Male; n (%)	61 (41.8)	4187 (75.3)	7239 (82.9)	4406 (77.8)			
Age median; IQR (years)	75.00 (67.00–81.75)	74.00 (67.00–80.00)	71.00 (63.00–77.00)	67.00 (59.00–74.00)	<0.001	0	0.35
Age					<0.001	0	0.38
<59; n (%)	16 (11.0)	549 (9.9)	1410 (16.1)	1440 (25.4)			
60–79; n (%)	83 (56.8)	3617 (65.0)	5864 (67.1)	3685 (65.1)			
>80; n (%)	47 (32.2)	1398 (25.1)	1462 (16.7)	538 (9.5)			
Height median; IQR (cm)	167.00 (160.00–174.75)	174.00 (167.00–180.00)	175.00 (169.00–180.00)	174.00 (168.00–180.00)	<0.001	4111 (16.9%)	0.37
Weight median; IQR (kg)	49.00 (44.00–53.00)	70.00 (63.00–75.00)	83.00 (77.00–90.00)	100.00 (92.00–110.00)	<0.001	3414 (14.1%)	2.88
BMI† median; IQR (kg/m²)	17.58 (16.65–18.07)	23.44 (22.04–24.34)	27.36 (26.20–28.41)	32.65 (31.14–35.06)	<0.001	4175 (17.2%)	3.42
Diabetes; n (%)	25 (17.4)	1153 (20.8)	2576 (29.5)	2346 (41.5)	<0.001	132 (0.5%)	0.31
Diabetes insulin usage; n (%)	16 (11.0)	523 (9.4)	1069 (12.3)	1153 (20.4)	<0.001	71 (0.30%)	0.16
Smoking status					<0.001	1443 (5.9%)	0.29
Current smoker; n (%)	48 (34.8)	1082 (20.4)	1387 (16.5)	937 (17.2)			
Non-smoker; n (%)	52 (37.7)	2166 (40.9)	3345 (39.7)	1895 (34.8)			
Previous smoker; n (%)	38 (27.5)	2052 (38.7)	3690 (43.8)	2614 (48.0)			
Extent of CTO					0.212	0	0.06
1 CTO; n (%)	122 (83.6)	4577 (82.3)	7221 (82.7)	4586 (81.0)			
2 CTO; n (%)	21 (14.4)	814 (14.6)	1237 (14.2)	871 (15.4)			
3 or more CTOs; n (%)	3 (2.1)	173 (3.1)	278 (3.2)	206 (3.6)			
Hypertension; n (%)	94 (65.7)	3778 (68.4)	6440 (74.1)	4613 (81.9)	<0.001	255 (1.1%)	0.21
Hyperlipidaemia; n (%)	80 (55.6)	3201 (58.0)	5397 (62.3)	3771 (67.1)	<0.001	326 (1.3%)	0.13
Previous MI; n (%)	50 (35.5)	1674 (30.7)	2700 (31.5)	1940 (34.9)	<0.001	669 (2.8%)	0.06
Indication for angiography					<0.001	111 (0.5%)	0.32
Stable CAD; n (%)	16 (11.0)	1368 (24.7)	2677 (30.8)	1631 (28.9)			
Unstable CAD/NSTEMI; n (%)	70 (47.9)	2340 (42.3)	3588 (41.3)	2442 (43.3)			
STEMI; n (%)	25 (17.1)	789 (14.3)	1100 (12.6)	644 (11.4)			
Cardiac arrest without STEMI; n (%)	0 (0.0)	68 (1.2)	81 (0.9)	68 (1.2)			
Cardiac arrest with STEMI; n (%)	5 (3.4)	27 (0.5)	48 (0.6)	32 (0.6)			
Other; n (%)	30 (20.5)	940 (17.0)	1202 (13.8)	823 (14.6)			
CCS class					0.360	18 (0.07%)*	0.14
I; n (%)	2 (12.5)	197 (14.4)	349 (13.1)	212 (13.0)			
II; n (%)	8 (50.0)	742 (54.4)	1495 (55.9)	853 (52.3)			
III; n (%)	6 (37.5)	414 (30.3)	798 (29.8)	544 (33.4)			
IV; n (%)	0 (0.0)	12 (0.9)	32 (1.2)	21 (1.3)			
eGFR median; IQR (mL/min/1.73 m²)	77.55 (58.50–89.70)	75.30 (58.32–87.30)	77.20 (61.30–89.20)	78.50 (60.30–91.70)	<0.001	5170 (21.3%)	0.08

* Calculated within the ‘stable CAD’ group only.

† BMI was missing for 4175 observations.

BMI, body mass index; CAD, coronary artery disease; CCS, Canadian Cardiovascular Society; CTO, chronic total occlusion; DM, diabetes mellitus; eGFR, estimated glomerular filtration rate; MI, myocardial infarction; n, sample size; NSTEMI, non-STEMI; SMD, standardised mean difference; STEMI, ST-elevation myocardial infarction.

### Statistical analysis

- Main analyses: Continuous variables are presented as medians with IQR and categorical variables as counts and percentages. Between-group comparisons used Wilcoxon rank-sum or Kruskal-Wallis tests for continuous variables and χ^2^ or Fisher’s exact tests for categorical variables. Baseline balance was quantified using standardised mean differences. Time-to-event analyses used Kaplan-Meier estimators with log-rank tests. Multivariable Cox proportional hazards models estimated HRs with 95% CIs, adjusting for prespecified covariates listed above.
- Missing data: Addressed by multiple imputation as described; pooled estimates are reported. Complete-case analyses were performed as sensitivity analyses.
- Loss to follow-up: Mortality follow-up was virtually complete through national linkage; loss to follow-up was not expected.
- Sensitivity analyses: Key analyses were repeated using complete cases and displayed imputed Kaplan-Meier curves to evaluate robustness (online supplemental figure S1 and S2). Proportional hazards assumptions were evaluated using Schoenfeld residuals and log-minus-log plots. All analyses were performed using Visual Studio Code (V.1.102.0; Microsoft, Redmond, Washington, USA) with the R kernel (V.4.4.1; R Foundation for Statistical Computing, Vienna, Austria). Survival, survminer, mice, rms, tableone and naniar packages were used for survival analysis and data visualisation. Statistical significance was defined as a two-sided p<0.05.

### Patient and public involvement

Patients and/or the public were not involved in the design, conduct, reporting or dissemination of this research.

## Results

### Baseline characteristics

#### Stratification by DM status

The baseline characteristics of the study population stratified by DM status are summarised in table 1. Of the 24 284 patients with CTO included in the study, DM status was missing for 132 patients. The analysis is based, therefore, on 24 152 patients, of whom 7352 (30.3%) were classified as having DM and 16 800 (69.7%) as non-DM.

In the study, the proportion of women with CTO was higher in patients with DM compared with patients without DM (22.6% vs 20.7%). Among males, 77.4% had DM, and 79.3% did not. The gender distribution between groups was statistically significant (p=0.002). DM patients had significantly lower eGFR compared with patients without diabetes (73.60 mL/min/1.73 m^2^ vs 77.90 mL/min/1.73 m^2^, p<0.001). Patients with DM exhibited significantly higher BMI compared with patients without DM (median: 28.68 kg/m² vs 26.78 kg/m², p<0.001). Median age was comparable between the two groups (71.00 years vs 71.00 years, p=0.235). Among the 7352 patients with DM, 3331 (45.7%) were treated with insulin at the time of angiography (71 missing; 0.3%).

Comorbidities were more prevalent among patients with DM, including hypertension (88.5% vs 67.7%), hyperlipidaemia (77.0% vs 54.8%) and a history of MI (39.9% vs 28.8%) (all p<0.001). Differences were also observed in smoking history: previous smoking was more common among patients with DM (47.0% vs 41.3%, p<0.001), whereas current smoking was slightly less prevalent in the DM group (17.0% vs 18.8%, p<0.001).

The number of CTO lesions varied between groups, with single-vessel CTO being more frequent in patients without DM (83.4% vs 79.3%, p<0.001). Conversely, two-vessel CTO and ≥3-vessel CTO were more common in DM patients (16.9% and 3.8%, respectively, vs 13.6% and 3.0% in patients without DM, p<0.001).

Regarding angiography indications, unstable CAD/NSTEMI was more frequently observed among DM patients (43.6% vs 39.4%, p<0.001), whereas stable CAD and STEMI were slightly more common in the non-DM group (28.4% vs 27.1% and 14.8% vs 11.5%, respectively, p<0.001).

Symptom severity, measured by the Canadian Cardiovascular Society (CCS) classification, primarily used for stable CAD patients, was largely similar between groups, with a higher proportion of class III symptoms in DM patients (33.1% vs 29.6%).

#### Stratification by BMI categories

Baseline characteristics stratified by BMI categories are presented in table 2. Among the 24 284 patients in the CTO cohort, 4175 (17.2%) were excluded due to missing BMI values.

The largest subgroup was the overweight (43.4%). Women were most prevalent in the underweight category (58.2%) and least prevalent in the overweight group (17.1%) (p<0.001). Conversely, men were most represented in the obesity category (82.9%, p<0.001). Underweight patients were the oldest (median: 75.00 years), while obese patients were the youngest (median: 67.00 years, p<0.001). Kidney function, as reflected by eGFR, was highest in the obesity group (78.50 mL/min/1.73 m^2^) and lowest in the healthy-weight group (75.30 mL/min/1.73 m^2^) (p<0.001).

The prevalence of comorbidities such as diabetes, hypertension and hyperlipidaemia increased proportionally with BMI. These comorbidities were least frequent in the underweight group (DM: 17.4%, hypertension: 65.7%, hyperlipidaemia: 55.6%) and most frequent in the obese group (DM: 41.5%, hypertension: 81.9%, hyperlipidaemia: 67.1%) (p<0.001).

Insulin therapy among patients with diabetes also varied significantly across BMI categories: 11.0% in underweight, 9.4% in healthy-weight, 12.3% in overweight and 20.4% in obesity (p<0.001).

Smoking habits also varied by BMI category. Current smoking was most frequent among underweight patients (34.8%) and least common in the obese group (17.2%). Non-smoking was most prevalent in the healthy-weight group (40.9%).

The extent of CTO lesions did not differ significantly across BMI categories (p=0.212).

Indications for coronary angiography varied by BMI category. Underweight patients were more likely to present with unstable CAD or NSTEMI (47.9%), STEMI (17.1%) and cardiac arrest with STEMI (3.4%). Stable CAD was most frequent in the overweight group (30.8%), while unstable CAD/NSTEMI was most common in obese patients (43.3%).

Symptom severity by CCS classification revealed minimal differences across BMI categories. Class I symptoms were observed in similar proportions across all groups (12.5%–14.4%). Class III symptoms were more prevalent in the underweight group (37.5%), while class IV symptoms were rare but highest among obese patients (1.3%). Overall, no significant differences in CCS class distribution were observed between the groups (p=0.360).

### Distribution of CTO location by BMI and DM

**Figure 2:**
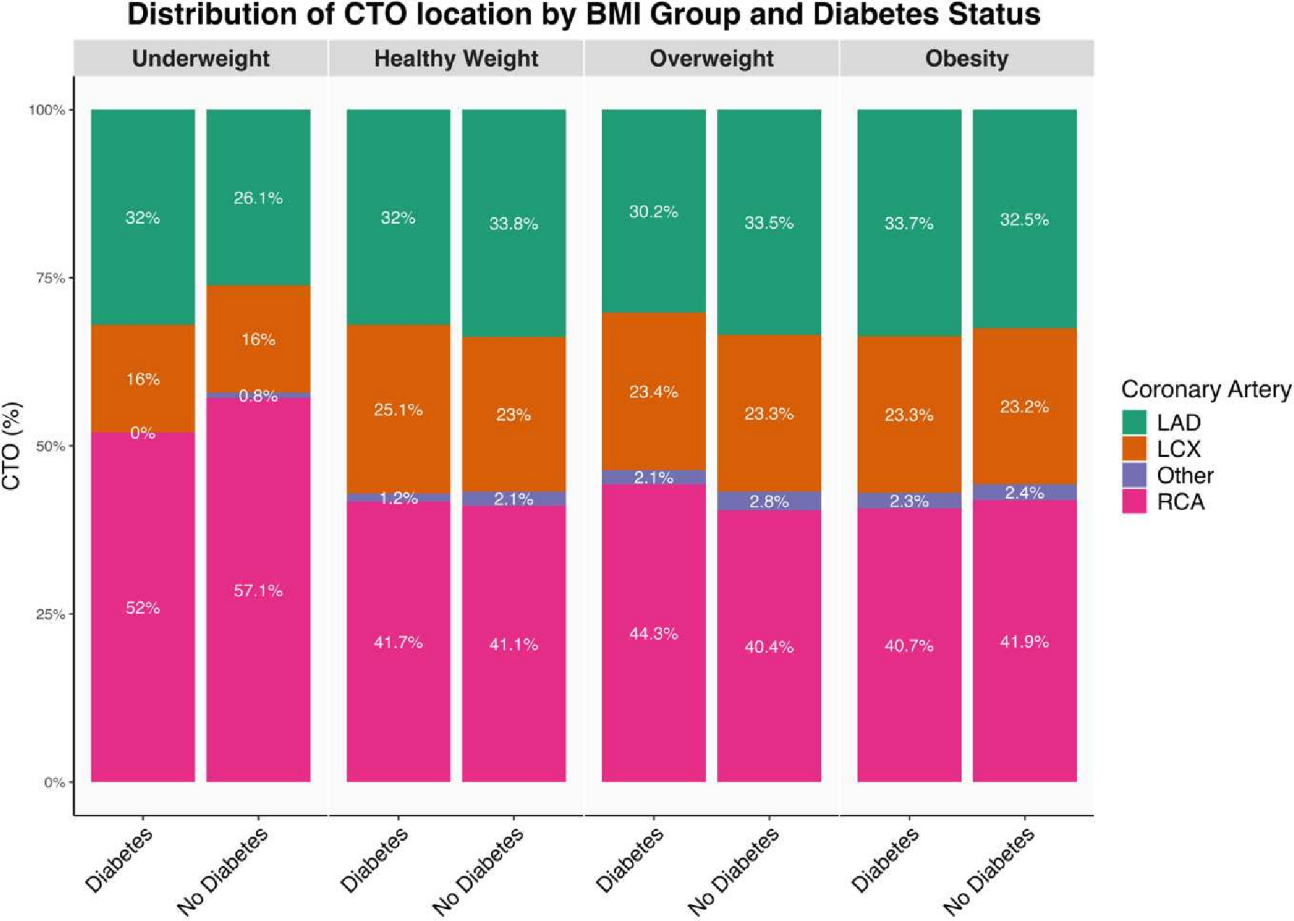
Distribution of CTO location by BMI group and DM status. Percentages are adjusted to sum precisely to 100% for each group. Other consist of left main artery, left posterior descending artery, posterior left artery from left, intermediate artery, right ventricular branch, septal artery. BMI, body mass index; CTO, chronic total occlusion; DM, diabetes mellitus; LAD, left anterior descending; LCX, left circumflex artery; RCA, right coronary artery.

Figure 2 shows the distribution of CTO locations by BMI categories and DM status. The right coronary artery was the most frequently affected vessel across all groups, with the highest prevalence observed in the underweight patients without DM (57.1%) and the lowest in overweight patients without DM (40.4%).

The left anterior descending artery was the second most common location, with its prevalence varying between 26.1% in underweight patients without DM to 33.8% in healthy-weight patients without DM.

Involvement of the left circumflex artery varied across BMI and DM strata. The highest prevalence was observed in healthy-weight patients with diabetes (25.1%), followed by overweight and obese groups (23.3%), whereas underweight patients had the lowest (16%).

Involvement of other arteries was minimal across all groups, ranging from no involvement to 2.8%.

### Survival analysis

**Figure 3:**
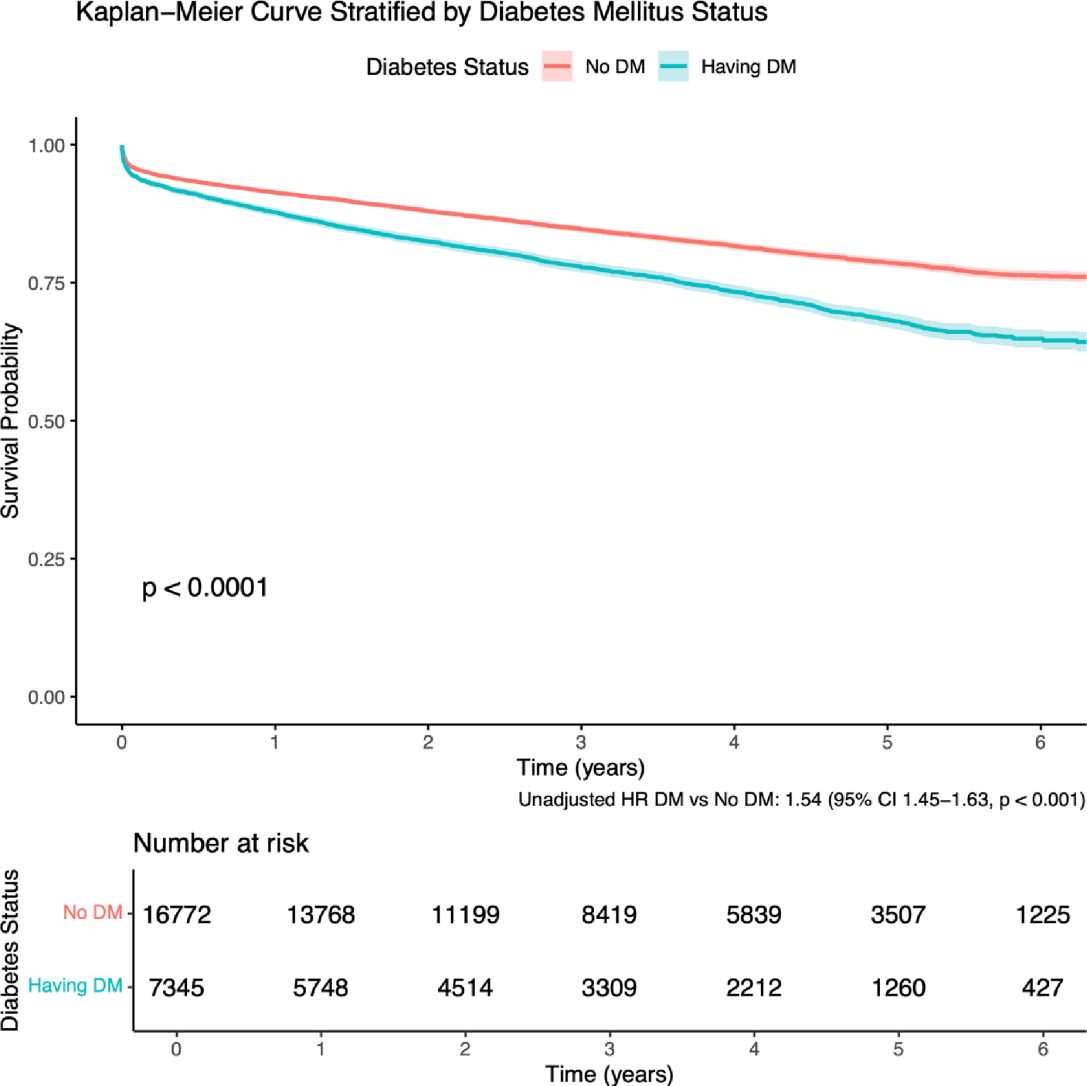
Kaplan-Meier survival curve stratified by DM status. Shaded areas represent 95% CI. 35 observations were not included in the analysis due to missing variable within the DM status cohort. DM, diabetes mellitus.

Kaplan-Meier survival analysis revealed statistically significant differences in survival based on DM status (figure 3). Patients with DM demonstrated lower survival probabilities compared with those without DM (unadjusted HR: 1.54, 95% CI: 1.45 to 1.63, p<0.001). The survival curves diverged early and continued to separate progressively over time. At the 5-year mark, the survival probability was 68.3% (95% CI: 66.94% to 69.76%) for patients with DM, compared with 78.7% (95% CI: 77.88% to 79.45%) for those without DM (log-rank p<0.0001).

**Figure 4:**
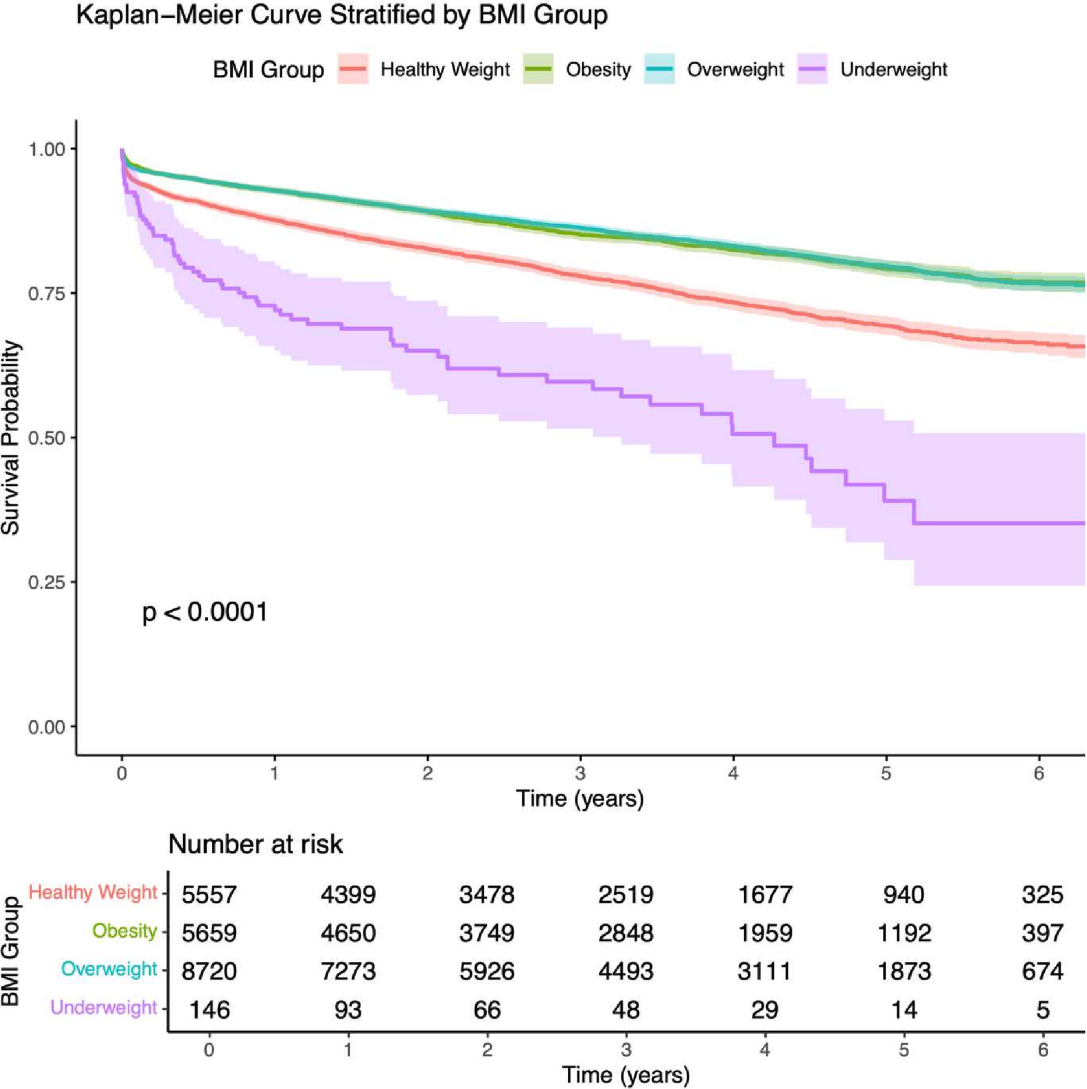
Kaplan-Meier survival curve stratified by BMI group. Shaded areas represent 95% CI. 27 observations were not included in the analysis due to missing variable within the BMI groups cohort. BMI, body mass index.

Survival outcomes also varied significantly across BMI categories (figure 4). Overweight and obese patients demonstrated the highest survival probabilities, while the underweight group had the lowest. Patients in the healthy-weight group showed intermediate survival rates. At the 5-year mark, the survival probability was 39.1% (95% CI: 28.81% to 52.95%) for the underweight group, 69.4% (95% CI: 67.81% to 70.96%) for the healthy-weight group, 79.7% (95% CI: 78.65% to 80.82%) for the overweight group and 79.3% (95% CI: 77.90% to 80.63%) for the obese group, demonstrating a statistically significant difference (log-rank p<0.0001).

As a sensitivity check, we repeated the 5-year Kaplan-Meier survival estimates in the first imputed dataset. The imputed curves again showed the highest survival among overweight and obese groups and the lowest among the underweight group, with the healthy-weight group in between. At 5 years, the survival probability was 37.2% (95% CI: 27.2% to 50.8%) for the underweight group, 68.7% (95% CI: 67.3% to 70.2%) for the healthy-weight group, 78.3% (95% CI: 77.3% to 79.3%) for the overweight group, and 78.0% (95% CI: 76.7% to 79.2%) for the obese group (log-rank p<0.0001). These estimates closely mirror those from the complete-case analysis, confirming the robustness of our findings.

### Multivariable Cox proportional hazards regression

**Figure 5:**
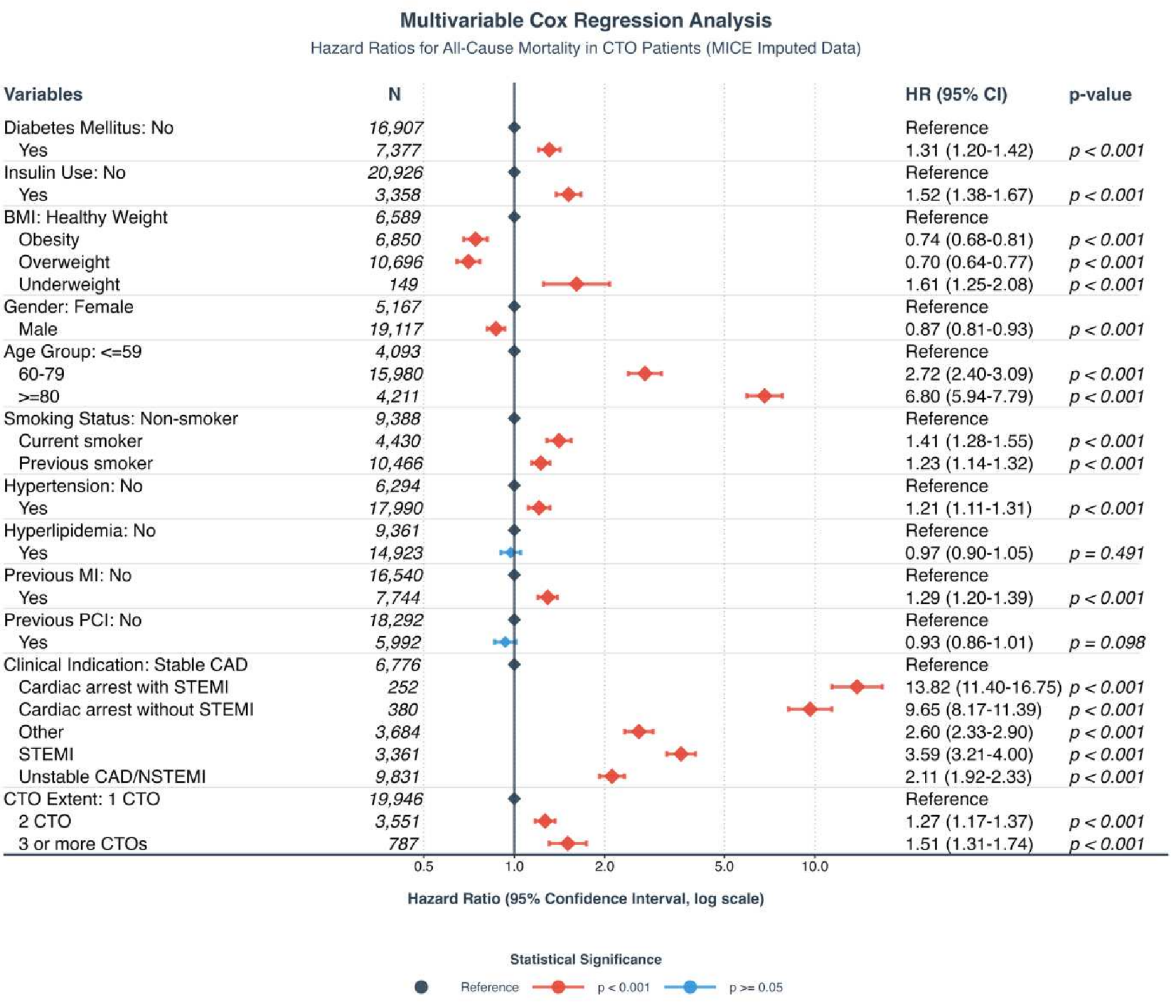
Multivariable Cox proportional hazards regression. Multivariate Imputation by Chained Equations (MICE) is a package in R programming language used for generating multiple imputations. BMI, body mass index; CAD, coronary artery disease; CTO, chronic total occlusion; MI, myocardial infarction; N, count of observations; NSTEMI, non-STEMI; PCI, percutaneous coronary intervention; STEMI, ST-elevation myocardial infarction.

Multivariable Cox regression pooled across five imputed datasets (m=5; n=24 284) identified both DM and BMI as independent predictors of mortality (figure 5). Compared with patients without diabetes, those with diabetes had a 31% higher risk of mortality (HR 1.31, 95% CI: 1.20 to 1.42; p<0.001). Among patients with diabetes, insulin use conferred an additional 52% increase in hazard compared with non-insulin users (HR 1.52, 95% CI: 1.38 to 1.67; p<0.001).

Across BMI categories, overweight and obese patients demonstrated significantly lower mortality risks compared with the healthy-weight group, with HRs of 0.70 (95% CI: 0.64 to 0.77; p<0.001) and 0.74 (95% CI: 0.68 to 0.81; p<0.001), respectively. In contrast, underweight patients exhibited the highest mortality risk (HR: 1.61, 95% CI: 1.25 to 2.08; p<0.001).

Other significant predictors of mortality included advanced age, smoking status, hypertension and previous MI. The highest mortality risk was observed in patients aged ≥80 years (HR: 6.80, 95% CI: 5.94 to 7.79; p<0.001), followed by patients aged 60–79 years (HR: 2.72, 95% CI: 2.40 to 3.09; p<0.001) compared with patients ≤59 years. Current smoking was associated with 41% higher hazard (HR: 1.41, 95% CI: 1.28 to 1.55; p<0.001), followed by previous smoking with a 23% higher hazard (HR: 1.23, 95% CI: 1.14 to 1.32; p<0.001) versus never-smoking.

Hypertension increased mortality risk by 21% (HR: 1.21, 95% CI: 1.11 to 1.31; p<0.001), whereas hyperlipidaemia was not significantly associated with mortality (HR: 0.97, 95% CI: 0.90 to 1.05; p=0.491). A history of MI conferred a 29% higher hazard (HR 1.29, 95% CI: 1.20 to 1.39; p<0.001); prior PCI did not significantly affect mortality (HR 0.93, 95% CI: 0.86 to 1.01; p=0.098).

Indications for angiography were also strong predictors of mortality. Using CAD as reference, patients presenting with cardiac arrest and STEMI as indication had the highest mortality risk (HR: 13.82, 95% CI: 11.40 to 16.75; p<0.001), followed by cardiac arrest without STEMI (HR: 9.65, 95% CI: 8.17 to 11.39; p<0.001), STEMI alone (HR: 3.59, 95% CI: 3.21 to 4.00; p<0.001), unstable CAD/NSTEMI (HR: 2.11, 95% CI: 1.92 to 2.33; p<0.001) and other indications (HR: 2.60, 95% CI: 2.33 to 2.90; p<0.001).

Finally, mortality risk increased with the number of CTOs. Patients with two-vessel CTO conferred a 27% higher hazard (HR: 1.27, 95% CI: 1.17 to 1.37; p<0.001) and those with three or more CTOs a 51% higher hazard (HR: 1.51, 95% CI: 1.31 to 1.74, p<0.001) compared with those with a single CTO.

### Continuous BMI relationship and mortality risk

**Figure 6:**
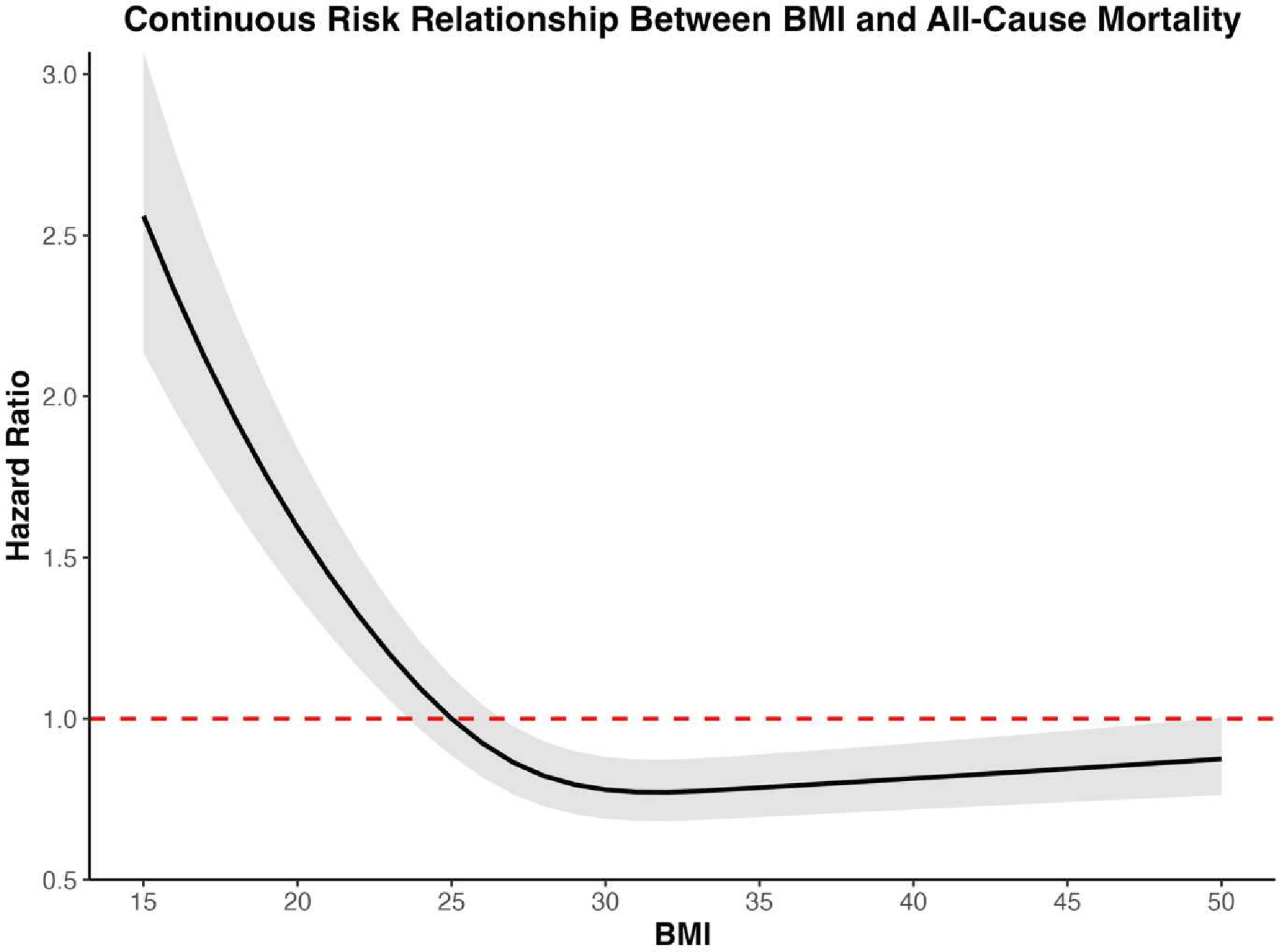
Continuous risk relationship between BMI and all-cause mortality. The model is adjusted to the included covariates: age group, gender, BMI, diabetes, insulin usage, smoking status, hypertension, hyperlipidaemia, previous MI and previous PCI. BMI, body mass index; MI, myocardial infarction; PCI, percutaneous coronary intervention.

The continuous association between BMI and all-cause mortality, as illustrated in figure 6, reveals a non-linear, asymmetric U-shaped association. Mortality hazard declined steeply from the underweight range, reaching its nadir at a BMI of 32 kg/m2 (HR: 0.77), within the obese category. Below 23 kg/m^2^, the hazard rose sharply, peaking at an HR of 2.56 at a BMI of 15 kg/m^2^. Above 35 kg/m^2^, mortality risk increased only modestly, remaining far lower than the risk observed in the underweight range.

The CIs expanded at extreme BMI values, reflecting increased uncertainty in risk estimates at both very low and very high BMI ranges.

## Discussion

In this nationwide cohort study of 24 284 patients with angiographically confirmed CTO, our analysis provides the most comprehensive evaluation to date of the prognostic influence of DM and BMI on long-term mortality.

The results revealed that DM was independently associated with increased all-cause mortality, with insulin therapy further elevating the hazard by 52%, highlighting a clear risk gradient linked to diabetes severity. Additionally, BMI demonstrated an asymmetric U-shaped association with survival, wherein overweight and mildly obese patients exhibited the most favourable survival outcomes, while underweight patients faced the highest mortality risk.

### Novel contributions

The study advances the current understanding with three novel contributions:

1. Generalisable, real-world risk estimates: By analysing the national SCAAR registry, we deliver robust risk estimates for CTO patients without prior CABG, reflecting the predominant patient population and overcoming the limitations of smaller, selected cohorts.
2. Precise continuous BMI spline in CTO: Through robust multiple imputation and restricted cubic spline modelling, we delineate a precise, non-linear relationship between BMI and mortality.
3. Hierarchical diabetes risk stratification: We quantify a stepwise mortality gradient in a CTO cohort, extending from non-diabetic patients to those with non-insulin-dependent and, highest of all, insulin-dependent diabetes.

### DM and mortality

DM is a well-established independent risk factor for cardiovascular diseases contributing to adverse outcomes. While its general impact is known, data specifically on the long-term impact of DM in CTO patients remains somewhat limited.^5 11 12^

Our study makes a significant contribution to this field. In a multivariable model, after adjusting for age, gender, BMI, smoking status, insulin usage, comorbidities (including hypertension, hyperlipidaemia and prior MI), previous PCI, indication for angiography and extent of CTO, we confirmed that DM was an independent predictor of mortality, with diabetic patients having a 31% higher risk compared with those without diabetes. Our observation that the survival curves for these groups diverged early and continued to separate over time. These findings are consistent with previous findings.^2 9 13^

Previous studies have also shown that DM significantly increases mortality, revascularisation rates and major adverse cardiac events (MACE) compared with patients without DM undergoing PCI for CTO. Even after successful PCI, DM remained a major risk factor. A possible explanation could be that patients with DM tend to have a higher number of lesions requiring management.^14^ The increased mortality risk in DM patients may stem from accelerated atherosclerosis, greater lesion complexity and microvascular dysfunction.^5^ DM is also associated with more severe and diffuse CAD, including higher rates of multivessel disease, smaller-calibre arteries and calcification. Incomplete revascularisation further exacerbates outcomes, increasing risks of recurrent ischaemia and major adverse cardiovascular events.^15^

Within the diabetic subgroup, insulin therapy was associated with a 52% higher mortality risk compared with patients with diabetes not on insulin, quantifying a clear stepwise gradient from no diabetes through non-insulin-dependent diabetes, and finally to insulin-dependent disease. This stepwise pattern emphasises the need for intensified risk management in this high-risk cohort. This mirrors findings from large PCI and CTO cohorts where insulin dependence predicted long-term mortality and MACE.^16^,^19^

Several pathophysiological mechanisms may explain these observations, including insulin-related weight gain, hypoglycaemia, and the proatherogenic state of hyperinsulinaemia.^20^

Importantly, this elevated risk likely reflects more advanced underlying disease and greater baseline metabolic disturbance, rather than a direct harmful effect of the insulin therapy itself. The distinction is critical because even after successful CTO revascularisation, insulin-dependent patients continue to face the highest residual risk.^20^,^24^

### BMI and mortality

This study revealed distinct differences in demographic, clinical and procedural characteristics based on BMI categories. Higher BMI was associated with more comorbidities such as diabetes, hypertension and hyperlipidaemia, whereas underweight patients had higher rates of smoking and more frequently experienced STEMI.

The findings confirmed the ‘obesity paradox’, with underweight patients experiencing the highest mortality risk and overweight/mildly obese individuals demonstrating the lowest risk.

Earlier small CTO-PCI studies observed survival benefits in overweight individuals but lacked power and generalisability, especially for obese subgroups.^6 25^ Among obese patients who underwent PCI, lower rates of clinical perforation were observed compared with overweight and healthy-weight patients, while technical success rates and major adverse cardiovascular and cerebrovascular events were comparable between obese and healthy-weight groups.^25^ By employing multiple imputation to address 17.2% missing BMI data and applying restricted cubic spline modelling, we generated a precise and generalisable BMI-mortality curve in CTO, confirming a steep increase in mortality below 23 kg/m^2^, a nadir at 32 kg/m^2^ and a modest rise above 35 kg/m^2^. This continuous analysis offers a clearer view of the obesity paradox, suggesting that the relationship between BMI and mortality follows a gradual risk gradient rather than distinct categories, which may aid clinical interpretation and risk stratification.

The mechanisms underlying the obesity paradox remain unclear and likely involve multiple factors. BMI’s inability to differentiate fat mass from lean mass may misclassify individuals. Additionally, early diagnosis and treatment in obese patients, better nutritional reserves, neurohormonal differences and higher cardiorespiratory fitness (CRF) may contribute to improved outcomes.^626^,^28^

CRF plays a crucial role in modulating the obesity paradox. Studies show that the paradox is most evident in individuals with low CRF, while it disappears in those with high CRF. Intentional weight loss, linked to lifestyle improvements and increased CRF, lowers mortality, whereas unintentional weight loss due to illness elevates risk. Misinterpretation of BMI as a risk factor without considering confounders such as chronic illness may distort conclusions.^7 26 27 29^

### The interplay of DM and BMI

The combined impact of diabetes severity and BMI on prognosis remains an underexplored area in patients with CTO. By evaluating these factors simultaneously, our findings indicate that the survival benefit associated with higher BMI persists in both non-diabetic and diabetic patients, suggesting that their effects on risk are likely additive rather than synergistic. Notably, our data hint at a possible shift in the BMI-mortality nadir with increasing diabetes severity, although this observation requires further validation. Such a trend may reflect that the metabolic disturbances associated with insulin-treated diabetes may weaken the protective effects typically linked to higher BMI.

### Strength and limitations

This study’s strengths include its large cohort size, comprehensive data from the SWEDEHEART registry, detailed baseline and procedural characteristics, and the robust handling of missing data via multiple imputation. These factors enabled robust analysis of DM and BMI’s impact on survival. Furthermore, SCAAR provides information on insulin use, offering valuable insights into diabetes management; however, the absence of data on DM duration, insulin duration and type of DM represents a limitation.

The principal limitation is the observational design, which precludes causal inference. Second, our reliance on BMI, without data on body composition or CRF represents a key limitation. Third, the absence of data on the duration of diabetes and glycaemic control (eg, haemoglobin A1c (HbA1c)) prevents a deeper analysis of metabolic status. Lastly, the cause of death was not adjudicated, limiting our ability to distinguish between cardiac and non-cardiac mortality. Additionally, residual confounding from unmeasured variables, such as socioeconomic status or medication adherence, cannot be fully excluded.

### Clinical implications and relevance

The findings underscore the importance of personalised management strategies in CTO patients:

1. A one-size-fits-all approach may be insufficient, particularly for those with DM or low BMI. Risk assessment should go beyond BMI to include other metrics such as CRF, fat distribution and comorbidities. Improving CRF through intentional weight loss and lifestyle modifications may offer better outcomes than weight loss alone.
2. Insulin-dependent diabetes should be recognised as a marker of very high residual risk. In line with current guidelines, management should emphasise evidence-based therapies beyond glucose control alone, such as SGLT2 inhibitors or GLP-1 receptor antagonists, which have demonstrated cardiovascular benefits irrespective of baseline HbA1c.^30^
3. The obesity paradox highlights the complexity of BMI as a prognostic factor. While higher BMI may confer survival advantages in certain populations, excess adiposity remains a significant risk factor for CVD progression. Clinical attention should shift towards patients with low or declining BMI, as this group appears to carry the highest risk. In high-risk CTO patients, preventing underweight and sarcopenia may be more important than aggressive weight loss in those with class I obesity. This reframes priorities without negating the benefits of guideline-recommended weight loss.
4. These findings point to a need for refined risk assessment tools. Future research should focus on improved methods for assessing body composition and CRF and explore their roles in CAD management.^25^,^2729^

## Conclusions

This nationwide study demonstrates that DM is independently associated with increased long-term mortality in CTO patients, with insulin therapy further amplifying this risk. BMI exhibits an asymmetric U-shaped relationship with mortality, showing lowest risk among overweight and mildly obese patients, and highest risk among underweight individuals. The observed ‘obesity paradox’ likely reflects unmeasured confounders rather than a true survival advantage of elevated BMI. These findings underscore the importance of individualised risk assessment and management strategies in CTO patients, particularly those with DM or low BMI.

## Supplementary material

10.1136/bmjopen-2025-100074 online supplemental file 1

## Data Availability

Data are available on reasonable request. Data may be obtained from a third party and are not publicly available.
