# Supplementary material for: Impact of diabetes mellitus and body mass index on long-term survival in chronic total occlusion patients: a nationwide cohort study from the SCAAR registry
Source: BMJ Open. 2025 Sep 17;15(9):e100074. doi: 10.1136/bmjopen-2025-100074 (PMC12458819; doi:10.1136/bmjopen-2025-100074)
Supplement: online supplemental file 1 [file bmjopen-15-9-s001.docx]

Figure S1 Kaplan-Meier Survival Curve Stratified by BMI Group Using Imputed Data (Sensitivity check)


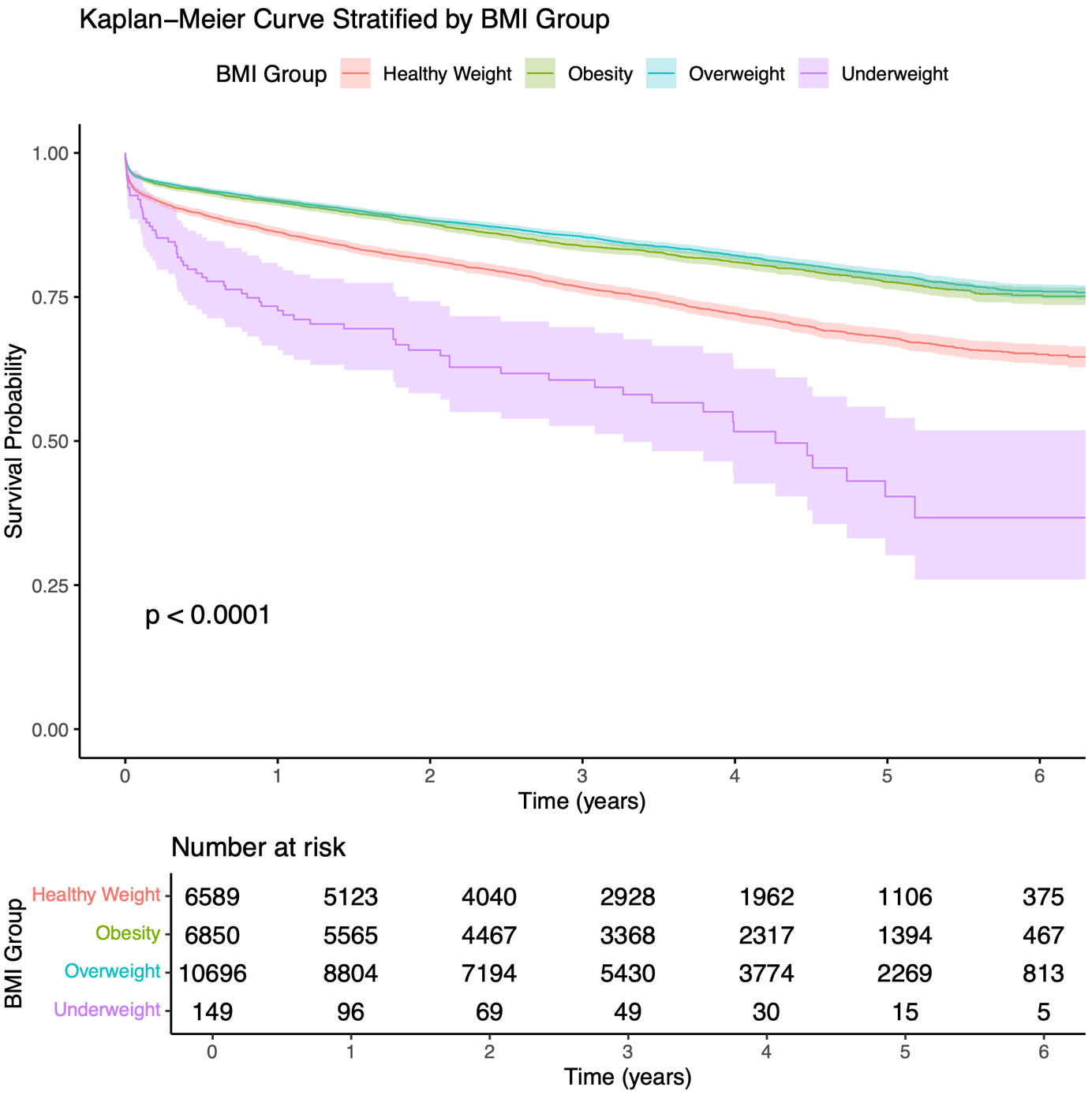


Shaded areas represent 95% Confidence Intervals (CI).
Total observations: 24,284

Figure S2 Multivariable Cox Proportional Hazard Regression of Non-imputed Data (Sensitivity with Complete-cases check)


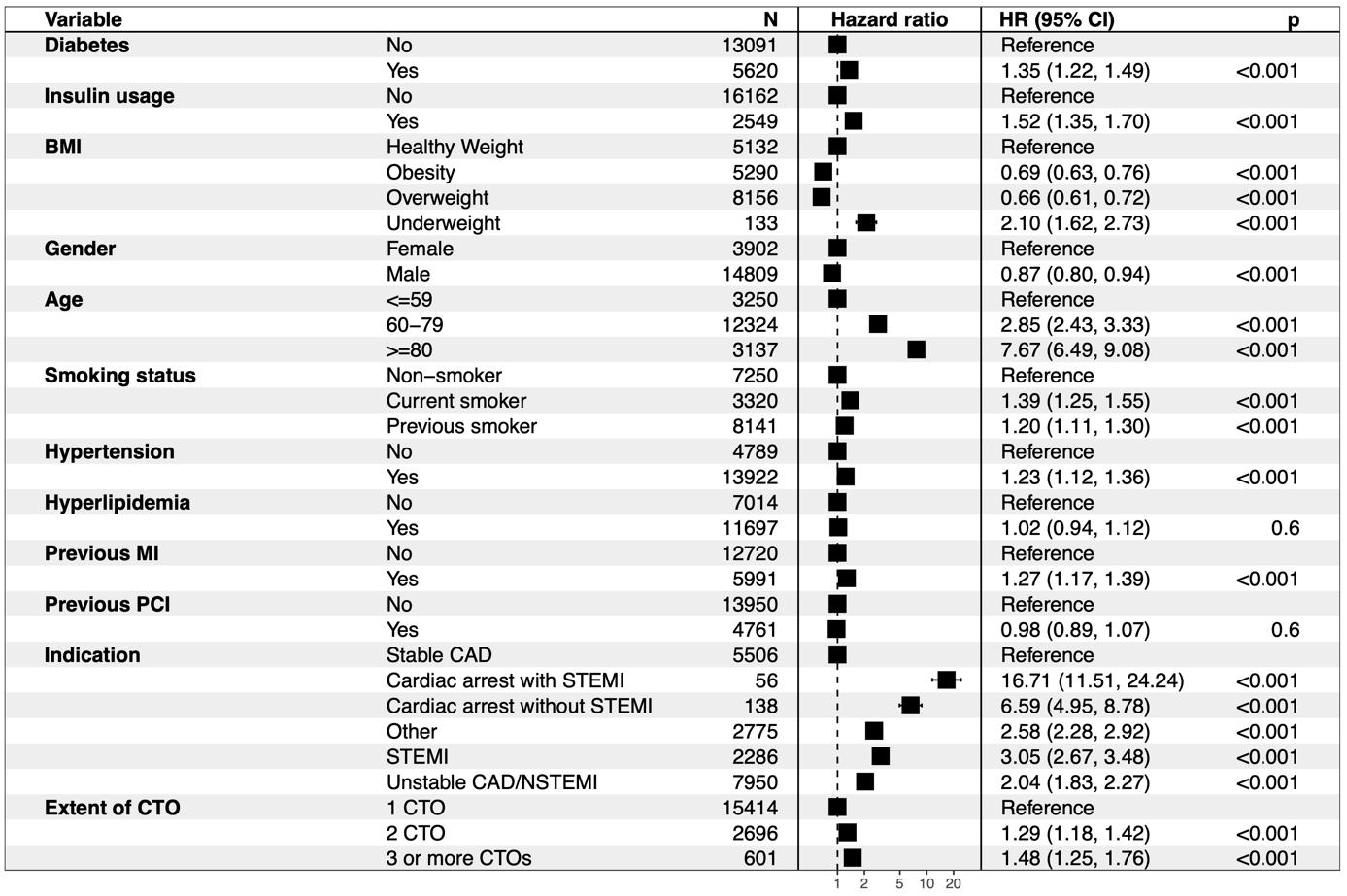


N = Numbers/Amount, HR = Hazard Ratio, CI = Confidence Interval, P = p-value, MI = Myocardial Infarction,
PCI = Percutaneous Coronary Intervention, CAD = Coronary Artery Disease,
(N)STEMI = (Non-)ST-Elevation Myocardial Infarction, CCS = Canadian Cardiovascular Society.
Same covariates as main model.
