# Supplementary material for: Impact of diabetes mellitus and body mass index on long-term survival in chronic total occlusion patients: a nationwide cohort study from the SCAAR registry
Source: BMJ Open. 2025 Sep 17;15(9):e100074. doi: 10.1136/bmjopen-2025-100074 (PMC12458819; doi:10.1136/bmjopen-2025-100074)
Supplement: Supplementary file 2 [file bmjopen-2025-100074.reviewer_comments.pdf]

PEER REVIEW HISTORY

BMJ Open publishes all reviews undertaken for accepted manuscripts. Reviewers are asked to complete a checklist review form and are provided with free text boxes to elaborate on their assessment. These free text comments are reproduced below.

ARTICLE DETAILS

Title (Provisional)

The Impact of Diabetes Mellitus and Body Mass Index on Long-Term Survival in Chronic Total Occlusion Patients: A Nationwide Cohort Study from the SCAAR Registry

Authors

Mohammed, Mohammed; sundström, joakim; Louca, Antros; Hellsen, Gustaf; Rawshani, Aidin; Olivecrona, Göran K; Mohammad, Moman Aladdin; Ioanes, Dan; Jensen, Ulf; Erlinge, David; Angerås , Oskar; Petursson, Petur; Myredal, Anna; Völz, Sebastian; Dworeck, Christian; Odenstedt, Jacob; Rawshani, Araz; Råmunddal, Truls

---

VERSION 1 - REVIEW

**Reviewer**	**1**
**Name**	**Luo, Miyang**
**Affiliation**	**Department of Epidemiology, Xiangya School of Public Health, Central South University**
**Date**	**16-May-2025**
**COI**	**None**

Overall, the manuscript addresses a meaningful topic with a large sample size, which is commendable. However, there are several concerns regarding the writing and presentation of data that need to be addressed.

1. The authors claim a J-shaped relationship between BMI and mortality. However, the results show that mortality increases with low BMI and decreases with high BMI, which does not align with the typical J-shaped pattern. A J-shaped relationship implies that both very low and very high values of a variable are associated with increased risk, with a nadir at intermediate values. This discrepancy needs to be clarified, and the terminology should be corrected to avoid confusion.

2. The manuscript reports high rates of missing data for some variables, which may impact the validity of the results. For instance, the missing data rate for BMI is concerning, given its central role in the study. The handling of missing data is not clearly described, and the

methods used should be more transparent. Specifically, the calculation of missing data rates for variables such as insulin treatment should be restricted to relevant subgroups (e.g., only among diabetic patients). The current approach may overestimate the missing rate and introduce bias. The authors should provide a detailed description of how missing data were handled and justify the chosen methods.

3. The phenomenon of the "obesity paradox" has been extensively reported in the literature. While the study provides additional evidence on this topic, the authors should highlight the unique contributions of their research. A more in-depth discussion of how this study advances the understanding of the relationship between BMI, DM, and mortality in CTO patients is warranted. The authors should emphasize any novel findings or insights that differentiate their work from previous studies.

**Reviewer**	**2**
**Name**	**Wiafe, Yaw Amo**
**Affiliation**	**Kwame Nkrumah University of Science and Technology**
**Date**	**11-Jul-2025**
**COI**	**None**

If available, please consider including additional information on duration of diabetes, glycemic control (e.g., HbA1c levels), and insulin use, as these may significantly influence prognosis.

VERSION 1 - AUTHOR RESPONSE

Reviewer 1: Prof. Miyang Lup

Comment 1: “The authors claim J-shaped relationship between BMI and mortality... This discrepancy needs to be clarified, and the terminology should be corrected...”

Response: We thank Professor Luo for this insightful observation. We agree that the relationship is not a classic J-shape. The mortality risk increases very steeply at low BMI values and only modestly at very high BMI values. Therefore, “asymmetric U-shaped” is more precise description. We have corrected this throughout the manuscript.

- The abstract now describes an “asymmetric U-shaped association”.
- The discussion uses the term “asymmetric U-shaped association”.
- The title for Figure 6 has been updated to “Continuous Risk Relationship Between BMI and All-cause Mortality” to avoid confusion, and the text refers to this as a non-linear, asymmetric U-shaped curve.

Comment 2: "The manuscript reports high rates of missing data for some variables... The handling of missing data is not clearly described... calculation of missing data rates for variables such as insulin treatment..."

Response: We appreciate the reviewer raising this critical point. We have taken several steps to ensure our handling of missing data is both robust and transparent.

- We have included a detailed subsection in the Methods titled "**Missing Data Assessment and Handling**" (Page 4). This section describes our systematic evaluation of missingness patterns and the use of **multiple imputation by chained equations (MICE)** to handle missing covariate data, thereby preserving the full analytical cohort and reducing selection bias.
- We performed a **complete-case analysis** as a sensitivity check to confirm the robustness of our findings.
- Regarding the missing rate for insulin usage, we have clarified that this was calculated only within the relevant subgroup of patients with diabetes. As stated in the Results (Page 5-6) among the 7,352 patients with DM, data on insulin treatment was missing for 71 individuals (0.3%).

Comment 3: "The phenomenon of the 'obesity paradox' has been extensively reported... the authors should highlight the unique contributions of their research... emphasize any novel findings or insights that differentiate their work..."

Response: We thank the reviewer for this valuable suggestion. To highlight the novelty of our work, we have added a dedicated subsection in the Discussion titled: "**Novel Contributions**" (Page 9). This section outlines three key advances, which include:

1. Providing generalizable, real-world risk estimates for CTO patients from a nationwide cohort.
2. Delineating a precise, non-linear BMI-mortality relationship through robust imputation and spline modeling.
3. Quantifying a hierarchical mortality risk based on diabetes severity and insulin use.

Reviewer 2: Dr. Yaw Amo Wiafe

Comment: "If available, please consider including additional information on duration of diabetes, glycemic control..."

Response: We thank Dr. Wiafe for this excellent point. We agree that these variables are of great prognostic importance.

- Acting on this valuable feedback, we have now incorporated a formal analysis of insulin use into our multivariable models. This new analysis revealed that insulin use is a powerful predictor of mortality. As now shown in our revised multivariable analysis, insulin use among diabetic patients conferred an additional 52% increase in mortality hazard compared to non-insulin users (HR 1.52, 95% CI: 1.38-1.67).
- Unfortunately, the SCAAR registry does not collect data on the duration of diabetes, glycemic control (e.g., HbA1c), or duration of insulin therapy. While this prevents a deeper analysis of metabolic status, we have acknowledged this as a key limitation in the "Strength and Limitation" subsection of the Discussion (Page 12).

We are confident that the revisions have addressed all concerns and have significantly improved the manuscript. We thank you again for your time and the opportunity to resubmit our work. We hope that the revised manuscript is now suitable for publication in BMJ Open.
